# The response to genetic merit for milk production in dairy cows differs by cow body weight

**DOI:** 10.3168/jdsc.2021-0115

**Published:** 2021-10-22

**Authors:** D.P. Berry, R.D. Evans

**Affiliations:** 1 Teagasc, Animal & Grassland Research and Innovation Centre, Moorepark, Fermoy P61 P302, Co. Cork, Ireland; 2 Irish Cattle Breeding Federation, Highfield House, Shinagh, Bandon P72 X050, Co. Cork, Ireland

## Abstract

Attention is increasing on both cow size and body weight (BW) as energy sinks and thus as contributors to differences in production efficiency among cows. What is not currently clear, however, is how cow BW affects the increase in yield per cow per unit increase in genetic merit for milk production. This void in knowledge was filled in the present study using BW data from 20,470 lactations on 16,980 Holstein-Friesian dairy cows stratified into 4 groups on BW adjusted for differences in parity, days in milk, and body condition score. Using linear mixed models that adjusted for nuisance factors, cow phenotypic milk production variables were regressed on estimates of parental average genetic merit for the respective trait within each stratum of BW defined within contemporary group; estimates of genetic merit were from the national genetic evaluations. Both the intercept and linear regression coefficients on genetic merit were compared across BW strata. The intercepts representing the mean phenotypic yield at a genetic merit of zero differed among BW strata; irrespective of yield trait, the least squares means yield per BW stratum increased numerically as cows got heavier, although not every stepwise increase in BW stratum was associated with significantly greater yield compared with the previous (lighter) stratum. Nonetheless, the yield of the cows in the lightest of the 4 strata was always less than that of the heaviest 2 strata; relative to the lightest stratum, cows in the heaviest BW stratum produced only 3 to 4% more yield. Furthermore, the association between phenotypic yield and its respective measures of genetic merit differed by BW stratum; the response to selection for each of the yield traits was 15 to 23% greater for the heaviest stratum of cows compared with their contemporaries in the lightest stratum. Although BW stratum was associated with mean fat and protein concentration after adjusting for differences in genetic merit for fat and protein concentration, the association did not differ by BW stratum for either fat or protein concentration. The effect of BW on efficiency should consider the association between BW and not only mean phenotypic yield at a given genetic merit, but also how the differences in yield diverge as genetic merit increases.

## Highlights

• Heavier cows yield more milk, fat, and protein.
• The association between genetic merit for milk yield and actual yield differs by BW.
• The association between genetic merit for milk composition and actual composition does not differ by BW.

## Graphical Abstract

**Figure:**
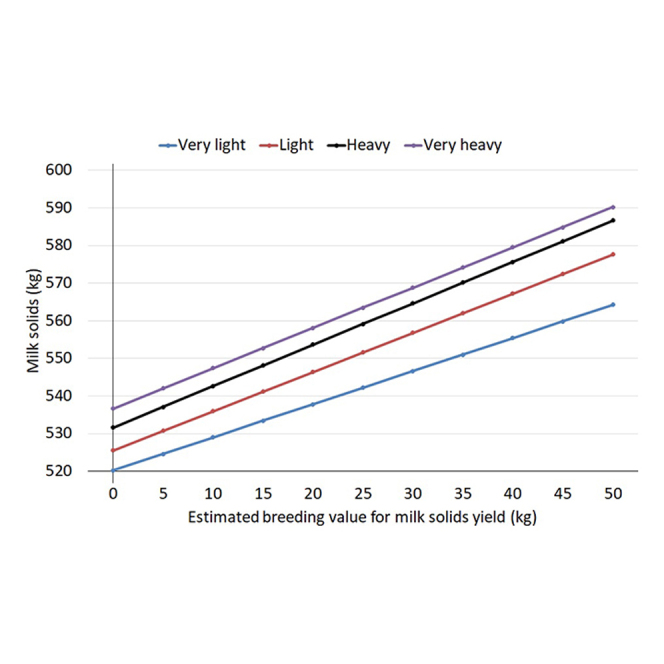
**Summary:** The larger the cow, the more she will eat on average. Increasing attention is focused on both cow size and body weight (BW) as energy sinks and thus as contributors to differences in efficiency of production among cows. Little is known, however, on how the response to selection for milk production is affected by cow BW, which, in turn, could negate some of the benefits in production efficiency of lighter cows. In addition to greater mean milk yield, fat yield, and protein yield for heavier cows, the response to selection for milk production was 15 to 23% greater in cows stratified as heavy versus contemporaries classified as light. Although mean differences in milk composition were evident between BW strata, the association between genetic merit for milk composition and actual observed milk composition did not differ by BW stratum.

In the pursuit of more efficient production, breeders and producers alike have begun focusing on energy sinks that do not directly contribute to increased output. One such trait is BW (O'Mara, 1996; NRC, 2001). Many dairy cow breeding goals now include cow BW (Cole and VanRaden, 2018) with a negative weighting factor; this negative weighting factor, however, does not necessarily imply a reduction in cow size because such breeding goals also seek higher milk output, which is often associated genetically with larger cows (Short and Lawlor, 1992; Berry et al., 2004; Vallimont et al., 2010). Nonetheless, there is a paucity of recent studies reporting the phenotypic association between BW and mean milk production, and also the response in milk output per unit change in genetic merit for milk output. This is especially true in large data sets, undoubtedly caused at least in part by a scarcity of nationally recorded BW data. This paucity of information is greater when considering grazing dairy cows where body size could be important to ensure the cow can fulfill a large proportion of her energy demands from grazed grass.

Using BW and milk production data from >2,500 lactation records of New Zealand grazing cows, Roche et al. (2007) documented higher yield in heavier cows; a similar conclusion was reported by Berry et al. (2007) using data from 9,886 lactations from Irish dairy cows. Neither study, however, investigated whether the response in milk production per unit change in genetic merit for milk production differed by cow BW. The same is true of studies in Finnish Ayrshire and Friesian cows (Hietanen and Ojala, 1995), which reported an association between cow BW and milk production but did not explore the association between response to selection for milk production and cow BW. The objective of the present study, therefore, was to use data from Irish spring-calving dairy cows to determine the association between BW and mean milk production at a constant genetic merit, and also the change in milk production per unit change in genetic merit for milk production. Genetic merit for milk production used in the present study was based on the entire population of milk-tested dairy cows in Ireland. The results will be useful in benchmarking the expected milk production of animals varying in both BW and genetic merit for milk production.

All phenotypic and genetic merit (i.e., EBV) data were extracted from the Irish Cattle Breeding Federation (http://www.icbf.com) database and are described in detail elsewhere (Berry and Kelleher 2021; Berry et al., 2021). A total of 34,476 BW and BCS observations recorded on the same day were available from 27,410 parities on 21,926 Irish Holstein-Friesian dairy cows calving between 2018 and 2020; all records were from 221 Irish dairy herds. All herds considered had to have data from at least 50 cows. Body weight was recorded using a weighing scale, and BCS was assessed on a 1 (emaciated) to 5 (obese) scale (Edmonson et al., 1989). Both traits were recorded either by producers or by 2 hired technicians. Only data from parities 1 to 15 were retained, which were subsequently collapsed into 1, 2, 3, 4, and 5+. Records were considered from cows where the BW and BCS observations were recorded in the same herd and where the cow had calved and resided in that herd for at least 100 d before data were recorded. A single BW and corresponding BCS record was retained per lactation nearest to 145 DIM but within 10 wk; 145 was chosen as the mid-lactation DIM based on the frequency distribution of the data available so as to maximize the number of records retained (Berry et al., 2021).

Only data from parities with a recorded 305-d milk yield, fat yield, and protein yield were retained; 305-d average milk fat and protein concentration were calculated from the respective yields. Fat plus protein yields were summed to generate a variable, hereafter referred to as milk solids yield (Berry et al., 2021). Obvious erroneous data were discarded. Estimated breeding values for all cows were based on the average EBVs of the respective sire and dam from the December 2017 national genetic evaluation; therefore, all cows retained had to have a known sire and dam, and the phenotypic data used in the present study were not included in the genetic merit estimates. The EBV for milk solids was calculated as the sum of the EBV for fat yield and the EBV for protein yield.

Animals were assigned to contemporary groups for use as a random effect in the subsequent statistical model but also for the creation of strata of cow BW. Contemporary group in the present study was defined as herd-year-season of calving based on the developed algorithm used for most of the national genetic evaluations in Ireland (Berry et al., 2013); only contemporary groups with at least 10 records were considered further, where the difference in calving date between the start and end of the contemporary group was no longer than 30 d. Following all edits, BW, BCS, and milk production data were available from 20,470 lactations from 16,980 cows in 657 contemporary groups from 144 Irish dairy herds. The proportion of records per parity was 0.23, 0.22, 0.18, 0.15, and 0.22 for parity 1 to 5+, respectively.

Before downstream analyses, BW records were adjusted using a fixed-effects multiple regression model that included parity, DIM, and BCS but also contemporary group; a 2-way interaction between parity and DIM was included but the association between BCS and BW did not differ by parity and therefore only the main effect of BCS was considered. Body weight was then adjusted to a mature cow equivalent, 145 d calved with a BCS of 2.75 (Berry et al., 2006); this adjusted BW was used in the further analyses. Four strata of (adjusted) BW were then generated within each contemporary group of calving separately, defined as very light, light, heavy, and very heavy with an equal number of cows per stratum where possible.

The association between BW stratum and each milk production trait was estimated using linear mixed models in PROC MIXED of SAS (SAS Institute Inc.), with contemporary group included as a random effect and cow included as a repeated effect. The dependent variables were 305-d milk yield, milk solids yield, fat yield, protein yield, fat concentration, and protein concentration. Parity was included as a nuisance fixed effect; the main effects of BW stratum (categorical variable) as well as genetic merit for the trait representing the dependent variable (continuous variable) were included in all models. The effect of a 2-way interaction between BW and genetic merit for each trait was also investigated; a 3-way interaction between genetic merit, BW stratum, and parity did not (*P* > 0.46) improve the fit to the data. The reference model solutions for each BW stratum at a genetic merit of zero (i.e., intercept) were for a mature cow.

Mean (standard error, SE) BW per parity from the model adjusting BW to a common DIM and BCS was 473 (0.75), 529 (0.74), 568 (0.80), 589 (0.87), and 611 (0.75) kg, for parity 1 to 5+, respectively; this implies that first-parity cows weigh, on average, 77% of mature cow weight (i.e., parity 5+) based on a mean DIM of 151 d and at a common BCS. This is similar to the values of 81 to 83% documented by Berry et al. (2011) and Buckley et al. (2000) in populations of Irish Holstein-Friesian cows not overlapping with the data used in the present study. This ratio is also identical to the value of 77% reported for the ratio of first-parity versus mature milk yield in Irish dairy cows (Berry and Ring, 2020).

The regression of BW on BCS from the model adjusting BW to a common parity, DIM, and BCS was 63.10 (SE = 1.19). This value is larger than the equivalent value of 39 to 50 kg of BW per unit of BCS (also on the 1 to 5 scale) when BW was regressed on BCS measures in Irish multiparous dairy cows assessed between 51 and 300 DIM (Berry et al., 2011). This difference is not due to a scaling effect of BW because the mean BW per parity in the study of Berry et al. (2011) was only approximately 20 kg heavier than the corresponding BW of the cows in the present study. What this relatively large contribution of BCS to differences in BW suggests, nonetheless, is that inter-animal differences in BCS do need to be accounted for when reporting BW values.

The raw mean BW and genetic merit performance statistics for different strata of BW are given in Table 1; the mean Holstein proportion of cows from the lightest to the heaviest stratum was 0.93, 0.94, 0.95, and 0.96, respectively. Cows in the heaviest BW stratum were 103 kg heavier (102 kg after adjusting for differences in parity, BCS, and DIM) than those in the lightest stratum, representing a difference of 19% relative to the mean of the entire population (552 kg). Nonetheless, as evidenced by the estimated standard deviations for BW per stratum, overlap in BW did exist between strata. The same was true for the measures of genetic merit per strata, which, of course, are also regressed toward zero as part of the genetic evaluation. Mean EBV for BW increased with stratum. A mean difference of 15 kg in parental average EBV for live-weight existed between the lightest and heaviest strata, implying that only a proportion of the phenotypic difference was actually due to a difference in parental average EBV. What is not clear, however, is whether the phenotypically lighter cows received a negative Mendelian sampling effect, with the heavier cows receiving a positive Mendelian sampling effect or, in fact, the observed phenotypic differences between strata were due to a legacy management effect contributing to undergrown or overgrown animals. However, 90% of the cows in the present study were born in the herd they produced in and thus are likely to have received similar heifer management. Limiting the data set to just these cows did not alter the study conclusions. Moreover, the mean difference, in genetic standard deviation units, for the EBV of animals in the lightest and heaviest BW strata was 0.45, 0.36, 0.29, and 0.16 units for stature, chest width, body depth, and BCS, respectively, signifying that animals in the heavier stratum were expected to be genetically taller, wider, and deeper with more body condition.

**Table 1:** Number of records (N), raw mean (SD) BW, and adjusted BW (BW_adj^1^) as well as mean (SD) EBV for BW, milk yields, and concentrations by BW stratum

BW stratum	N	BW (kg)	BW_adj (kg)	EBV						
				BW (kg)	Milk yield (kg)	Milk solids yield (kg)	Fat yield (kg)	Protein yield (kg)	Fat percent (%)	Protein percent (%)
Very light	5,078	504 (57.5)	548 (35.3)	−12 (19.6)	88.6 (236.10)	28.2 (16.97)	16.5 (10.71)	11.7 (7.78)	0.22 (0.181)	0.15 (0.085)
Light	5,134	535 (57.9)	586 (29.6)	−7 (−17.9)	105.4 (232.15)	29.4 (16.14)	17.0 (10.33)	12.3 (7.36)	0.22 (0.179)	0.15 (0.084)
Heavy	5,102	563 (57.9)	612 (29.9)	−2 (18.0)	119.6 (238.83)	30.1 (16.31)	17.3 (10.36)	12.7 (7.51)	0.21 (0.180)	0.15 (0.085)
Very heavy	5,156	607 (62.7)	651 (37.0)	3 (17.3)	136.5 (237.57)	30.1 (15.86)	17.2 (10.10)	13.0 (7.34)	0.20 (0.181)	0.14 (0.088)

1 BW adjusted for parity, DIM, and BCS.

In most instances, mean genetic merit for all yield traits increased with each stratum increase in BW. There was, however, minimal difference between strata in mean genetic merit for fat and protein concentration. The observed increasing genetic merit for milk production with increasing cow phenotypic BW (Table 1) is not unsurprising, given the known, albeit often weak, genetic correlations between milk yield and BW (Ahlborn and Dempfle, 1992; Berry et al., 2003). Some studies in dairy cattle have actually reported negative genetic correlations between BW and milk production (Vallimont et al., 2010), although the correlations can sometimes be breed specific (Hietanen and Ojala, 1995). Given the negative genetic correlations between milk production and BCS (Pryce et al., 2001; Veerkamp et al., 2001; Berry et al., 2003), some of the apparent discrepancies in the previously reported genetic correlations between BW and milk production may be due to the contribution of differences in BCS. Using the genetic correlations presented by Berry et al. (2003) among mid-lactation BW, BCS, and milk yield, the genetic correlation between mid-lactation BW and milk yield doubled in strength (from 0.18 to 0.37) when adjusted genetically for differences in BCS. This is more comparable to the present study, where BW was preadjusted to a common BCS. A similar conclusion was presented by Veerkamp and Brotherstone (1997) in that the genetic correlation between milk yield and BW in Holstein-Friesian cows changed from −0.09 to 0.29 once adjusted for differences in BCS. Of more interest in the present study, though, which has not been previously investigated, is whether the BW of the animal influences the response to selection for milk production.

The intercept and slope coefficients for each phenotypic yield trait on its respective EBV is in Table 2. Across all data, the slope (SE) of the regression of phenotypic milk yield, milk solids yield, fat yield, and protein yield on its respective EBV was 1.20 (0.03), 1.04 (0.03), 1.01 (0.03), and 1.17 (0.03), respectively, although it differed by parity; for milk yield, for example, the linear regression coefficients were 0.84 and 1.31 in first- and third-parity cows, respectively. The regression coefficient (SE) of BW on its respective EBV was 1.09 (0.02).

**Table 2:** Regression coefficients for the intercept (SE) and slope (SE) of phenotypic value on the respective EBV for milk production traits for a mature (i.e., parity 5+) cow

BW stratum	Intercept				Slope			
	Milk yield (kg)	Milk solids (kg)	Fat yield (kg)	Protein yield (kg)	Milk yield (kg)	Milk solids (kg)	Fat yield (kg)	Protein yield (kg)
Very light	6,741^a^ (31.6)	520.2^a^ (2.71)	280.8^a^ (1.48)	238.6^a^ (1.28)	1.07^a^ (0.050)	0.88^a^ (0.050)	0.87^a^ (0.046)	1.00^a^ (0.051)
Light	6,863^b^ (31.6)	525.6^ab^ (2.74)	283.3^ab^ (1.49)	241.7^a^ (1.29)	1.20^ab^ (0.049)	1.04^b^ (0.051)	1.00^b^ (0.046)	1.17^b^ (0.052)
Heavy	6,965^c^ (31.7)	531.6^bc^ (2.76)	285.6^bc^ (1.50)	246.1^b^ (1.30)	1.12^a^ (0.048)	1.10^b^ (0.051)	1.10^b^ (0.047)	1.11^ab^ (0.052)
Very heavy	6,999^c^ (31.9)	536.7^c^ (2.80)	289.3^c^ (1.52)	246.8^b^ (1.32)	1.29^b^ (0.049)	1.07^b^ (0.053)	1.08^b^ (0.049)	1.23^b^ (0.054)

a–c Values with different superscripts within a column signify a difference (*P* < 0.05).

For all yield traits, mean phenotypic yield differed (*P* < 0.001) between BW strata at a genetic merit of zero (i.e., the intercept); moreover, the association between phenotypic yield and its respective EBV differed (*P* < 0.001) by BW stratum. Irrespective of the yield trait, the mean yield per BW stratum increased consistently as cows got heavier, although not every stepwise increase in BW stratum was associated with a greater yield. Nonetheless, the yield of the cows in the lightest of the 4 strata was always less (*P* < 0.05) than that of the heaviest 2 BW strata. Relative to the lightest stratum, cows in the heaviest BW stratum produced only 3 to 4% more. There was no consistent trend across BW strata in the response in yield per unit change in the respective EBV. Nevertheless, the response in milk yield, milk solids yield, fat yield, and protein yield per unit change in the respective EBV was 21, 22, 15, and 23% greater, respectively, in the heaviest BW stratum versus the lightest BW stratum. This would manifest itself, therefore, as the phenotypic yield per BW stratum diverging as genetic merit for the yield traits increased. For example, based on the genetic standard deviation for milk yield in Ireland of 383 kg (http://www.icbf.com; accessed July 2021), the mean phenotypic difference in milk yield between the lightest and heaviest BW stratum is expected to be, on average, 4, 5, and 6% when the EBV for milk yield is 0, 1, or 2 genetic standard deviations above the mean. The mean difference in BW between both strata was 2.07 phenotypic standard deviations in BW, based on data in the present study adjusted for parity, DIM, and BCS. This difference therefore represents the average of the lightest and heaviest 36% of animals; thus, greater differences in milk yield would be expected in animals of more extreme BW, assuming the model solutions in the present study extrapolate out. Moreover, the results in the present study relate only to Irish grazing dairy cows; different results in other populations may materialize due to differences in the mean and variability in BW as well as milk yield. It is also unclear whether the associations observed in the present study would transfer to cows fed a more energy-rich diet in confinement production systems. Body size or weight is likely to influence intake capacity, a feature particularly important in grazing production systems, where capacity may be limited, especially in early lactation. Moreover, the generated strata could also reflect differences in Holstein proportion (relative to Friesian) and the impact this could have on associations with performance; this does not seem to be the case in the present study. Roche et al. (2007) associated milk production with BW in New Zealand grazing dairy cows. Using their model solutions for Holstein-Friesians, the expected difference in FCM yield between cows differing at nadir by 102 kg (mean difference in the present study between the lightest and heaviest stratum) was 11% of the mean; the associations reported by Roche et al. (2007), however, were not adjusted for differences in BCS. In a population of almost 10,000 lactations from (predominantly grazing) Irish Holstein-Friesian dairy cows, Berry et al. (2007) reported a nonlinear association between nadir BW and 305-d milk production; the difference in 305-d milk yield between cow BW values reflecting the mean BW of the lightest and heaviest strata in the present study (i.e., 102 kg) represented 3.3% of the mean 305-d milk yield in their study, similar to that in the present study, despite there being no animals in common between the analyses.

Although BW stratum was associated with mean fat (*P* < 0.001) and protein (*P* < 0.05) concentration after adjusting for differences in EBV for fat and protein concentration (i.e., the model intercept), the association between concentration and genetic merit did not differ by BW stratum for either fat (*P* = 0.31) or protein (*P* = 0.94) concentration. The regression of fat concentration on EBV for fat concentration was 1.19 (SE = 0.02), whereas that for protein concentration was 1.24 (SE = 0.02), both of which were greater (*P* < 0.001) than the expected value of 1. The least squares means for fat concentration (after adjusting for parity and contemporary group and the covariance among records within cow) from the lightest to heaviest BW strata were 4.381, 4.360, 4.348, and 4.347%, respectively, with standard errors of all estimates being 0.0098. The least squares means for protein concentration (after adjusting for parity and contemporary group and the covariance among records within cow) from the lightest to heaviest BW strata was 3.693, 3.695, 3.698, and 3.702%, respectively, with the standard errors of all estimates being 0.0044. Hence, although statistically significant differences in milk concentration were detected between BW strata, the differences were biologically very small, especially for protein concentration. Based on a population of New Zealand dairy cows, Roche et al. (2007) failed to detect an association between BW at calving and milk fat concentration, although a positive association was detected with BW at nadir in both Holstein-Friesians and Jersey cows. In that study, every 10-kg difference in nadir BW was associated with a 0.0076-percentage-unit increase in fat concentration in Holstein-Friesians, with the same difference in BW at nadir being associated with a 0.0065-percentage-unit increase in protein concentration. Similarly, Berry et al. (2007) failed to detect an association between each 10-kg increase in BW with 305-d fat concentration, although there was a weak association with protein concentration of just a 0.00012-percentage-unit increase in a population of Irish Holstein-Friesian cows independent of that used in the present study.

Given the population parameters reported in the present study, or any other population where such information exists, it is possible to quantify the proportion of the energy required that can be apportioned to lactation versus maintenance (for illustrative purposes, only these 2 energy sinks was considered here). The net energy of maintenance (NE_M_) and lactation (NE_L_) were calculated as follows (O'Mara, 1996):

$$NE_{M}=1.2\cdot \left[1.4+\left(\frac{BW}{100}\right)\right],$$

where the 1.2 factor was used to account for activity in grazing dairy cows, and NE_L_ = (0.0054·FC + 0.0031·PC + 0.0028·LC – 0.015) milk yield, where FC, PC, and LC are the fat, protein, and lactose concentration, respectively, in grams per kilogram; lactose concentration in the calculations here was assumed constant across BW strata. The daily NE_M_ for the lightest and heaviest BW strata in the present study (Table 1) was 5.63 unité fourragère lait (**UFL**) and 6.37 UFL, respectively. Using the intercept and regression solutions per stratum from the multiple regression model regressing phenotypic yield on its respective EBV, the daily NE_L_ for the lightest and heaviest stratum at a genetic merit of zero were 9.79 and 10.12 UFL. This implies that, in the lightest and heaviest strata, the proportion of energy demand (from these 2 sources) attributable to NE_L_ was 0.634 and 0.614, respectively. The proportion attributable to NE_L_ was always greater for the lighter BW stratum until the genetic merit of the cow was 34.5 standard deviations greater; despite this being unrealistic, it would also have to assume that the BW of the cow did not change. Hence, based on the sample population of commercial grazing dairy cows used in the present study, the relatively simple calculations (e.g., assuming no difference in net feed efficiency) of daily efficiency of converting feed ingested to lactation output was greatest for the lighter cow; not considered here, however, is any potential effect of BW on cow longevity and its impact on lifetime efficiency. Information is generally lacking on the association between cow BW (stratified within herd) and longevity, especially in grazing production systems where feed is generally limited.

The implications of the results from the present study are that expected phenotypic differences among animals (i.e., dams or sires) based on their respective estimates of genetic merit (Ring et al., 2021) would need to be rescaled based on the BW of the cow. The extent of rescaling would be a function of the BW of the cow herself. A shortcoming of the present study is the inability to fully disentangle the contribution of genetics versus management to the divergence in cow BW. Although differences in mean parental EBV for BW existed between the BW strata, the Mendelian sample variance, which is half the additive genetic variance, could not be captured in the present study. Hence, quantification of the extent of the actual genetic differences among strata was not possible. Nonetheless, when the data were limited to just cows that were born in the same herd where they produced (i.e., likely to be managed the same as contemporaries), the results did not alter the conclusions. A well-designed controlled experiment is required to properly disentangle genetic from management influences on cow BW and its effect on subsequent milk production performance.
