# Erratum to “The response to genetic merit for milk production in dairy cows differs by cow body weight” (JDS Commun. 3:32–37)

**DOI:** 10.3168/jdsc.2022-3-5-377

**Published:** 2022-09-12

**Authors:** D.P. Berry, R.D. Evans

The animal ethics statement was missing from this paper. The following statement should have been included: **No animals were used in this study, and ethical approval for the use of animals was thus deemed unnecessary**.
